# The delivery of specialist spinal cord injury services in Queensland and the potential for telehealth

**DOI:** 10.1186/s12913-016-1256-2

**Published:** 2016-01-25

**Authors:** Eileen van de Pol, Karen Lucas, Timothy Geraghty, Kiley Pershouse, Sandra Harding, Sridhar Atresh, Annemarie Wagemakers, Anthony C. Smith

**Affiliations:** 1 Health and Society, Department of Social Sciences, Wageningen University, Wageningen, The Netherlands; 2 PAH Telehealth Centre, Metro South Health, Princess Alexandra Hospital, Queensland, Australia; 3 Queensland Spinal Cord Injuries Service, Division of Rehabilitation, Princess Alexandra Hospital, Queensland, Australia; 4 Spinal Outreach Team, QLD Spinal Cord Injuries Service, Division of Rehabilitation, Princess Alexandra Hospital, Queensland, Australia; 5 Centre for Online Health, The University of Queensland, Queensland, Australia; 6 Princess Alexandra Hospital, Postal: PAH Telehealth Centre, Ground Floor, Main Building, Princess Alexandra Hospital, Woolloongabba, 4102 Australia

## Abstract

**Background:**

The Queensland Spinal Cord Injuries Service (QSCIS) is a statewide service in Brisbane at the Princess Alexandra Hospital (PAH). The QSCIS assists individuals with a spinal cord injury (SCI) through three services: the Spinal Injuries Unit (SIU), Transitional Rehabilitation Program (TRP) and the Spinal Outreach Team (SPOT). The aim of this study was to undertake a review of ambulatory services provided by the QSCIS (SIU and SPOT) to help identify where telehealth may potentially be useful.

**Methods:**

Profiling of patients with SCI in Queensland was achieved using database records containing referral data. Services provided by SIU Outpatient Clinics and the SPOT during a 6-year period (January 2008 – December 2013), were analysed. Using postcodes, we estimated distances between place of residence and Brisbane. We compared the general population of SCI patients with patients managed through SIU Outpatient Clinics and the SPOT.

**Results:**

During the 6-year period, 2073 patients were referred to the QSCIS (and living) at the time of the analysis. 74 % of all patients were male. The median age was 51y (IQR 39y-61y). About two-thirds of all patients lived within 200 km of Brisbane. 24 % of all patients registered with the QSCIS lived further than 200 km away from Brisbane.

7513 appointments were provided in the SIU outpatient clinic. 43,827 occasions of service were reported by the SPOT, including telephone consultations (66 %) and home visits (26 %). 72 outreach clinics were held in selected regional sites for up to 100 patients per year. 13 videoconference appointments reported.

90 % of all patients who attended the SIU outpatient clinic lived within 200 km of Brisbane. About two-thirds of patients who received a service from the SPOT lived within 200 km of Brisbane.

**Conclusion:**

Since one third of all patients registered with the QSCIS live at least 200 km away from Brisbane; it appears that these patients may not be accessing the same services as Brisbane based patients. Telehealth models of care, which promote better engagement with local health service providers (such as general practitioners, nurse practitioners and allied health professionals) could improve equity of access and reduce the need for extensive travel.

## Background

Queensland is Australia’s second largest and most decentralised state, covering a land area of 1,722,000 km^2^ (1,070,001 miles^2^). In Queensland, the vast geography and centralisation of specialist health services add to the challenges faced by patients living in regional, rural and remote areas [1]. People may need to travel very long distances as many specialist services are located in the capital city of Brisbane, in the south-east corner of the state. Equity of access to specialist services is an important issue for people all over the state however; it may be of even greater significance for people with severe disability for whom traveling long distances may be more challenging.

### Spinal injuries in Queensland

In Queensland, approximately 90 people each year suffer significant neurological loss secondary to an acute spinal cord injury (SCI) and require admission to the Spinal Injuries Unit (SIU) in Brisbane [2]. Motor vehicle accidents, falls, and sport are the most common causes of traumatic spinal cord injury [3]. An increasing number of admissions to the SIU in Brisbane are also related to non-traumatic causes of SCI including infection, transverse myelitis and prolapsed intervertebral discs. The average length of stay in the SIU is 2–3 months but some people require hospital admission for 6–12 months or longer due to the complexity of acute management, primary rehabilitation and discharge planning [4].

While most people with significant neurological deficit following SCI are transferred to the Spinal Injuries Unit at the Princess Alexandra Hospital (PAH) for initial management and primary rehabilitation almost all are discharged back to live in their local communities upon completion of their inpatient program and transitional rehabilitation program.

**Fig. 1:**
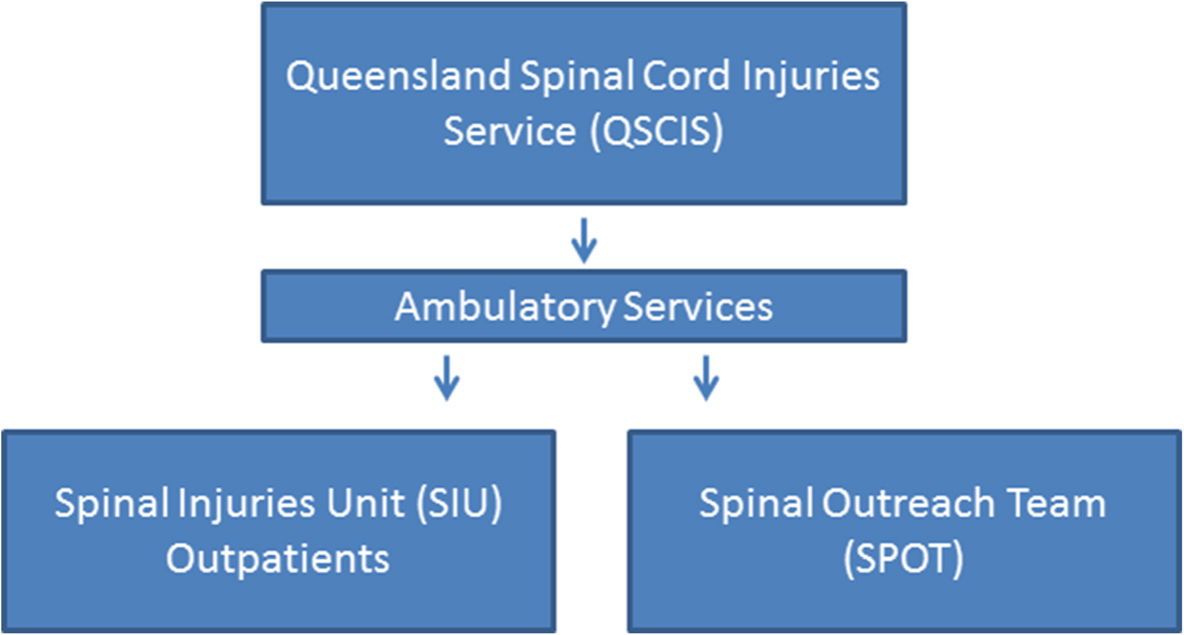
QSCIS – ambulatory services

Life-long follow-up for health maintenance, surveillance and management of complications is offered to all people with SCI in Queensland by the Queensland Spinal Cord Injuries Service (QSCIS), through the Spinal Outreach Team (SPOT) and SIU Outpatient Clinics [5], see Fig. 1.

### Queensland Spinal Cord Injuries Service (QSCIS)

The Queensland Spinal Cord Injuries Service is the state-wide, tertiary referral centre and only specialist service for the management of people with significant neurological deficit following spinal cord injury (SCI). It provides a comprehensive range of services for people with SCI through its three component services – the Spinal Injuries Unit (inpatient acute management and primary rehabilitation as well as life-long outpatient services), the Transitional Rehabilitation Program (TRP) (early discharge and transitional rehabilitation) and the Spinal Outreach Team (ongoing rehabilitation, outreach and long term follow-up ) [2]. The need for life long monitoring to more effectively address prevention of secondary conditions and ameliorate decline in function is recognised as people with SCI become older [6].

SIU Outpatient Clinics offer a range of specialist and general rehabilitation clinics including;

- General SCI medical / rehabilitation (Rehabilitation Medicine)
- Pressure Injury / Plastics (Multi-disciplinary team (MDT) including Plastic Surgeons and Rehabilitation Medicine)
- Neuropath Urology (Urology)
- Spasticity (Rehabilitation Medicine and MDT including physiotherapy and occupational therapy from SPOT as required)
- Musculoskeletal (Rehabilitation Medicine)
- Sex and Fertility Clinic (Rehabilitation Medicine)
- Adult Spina Bifida Clinic (Rehabilitation Medicine)

The Spinal Outreach Team, which commenced in late 1995, is a goal directed allied health and nursing consultancy and early intervention service and provides interdisciplinary management of spinal cord injury (SCI) along the lifespan for people with SCI, their significant others and for health professionals and service providers throughout Queensland [5]. The nature of the intervention and the method of implementing depend on the presenting problem and its complexity, other health and support services the client can access, the client’s location and maximising the resources of the team. Hence methodologies such as telephone and email are most frequently used to support individuals with SCI and their service providers. In addition, face to face visiting in a client’s home or other community location such as local hospital or workplace is utilised. On a daily basis the service can visit clients within a 200 km radius of Brisbane and regional visits are scheduled to clients living outside 200kms of Brisbane throughout the year. Supporting local health professionals in their intervention with people with SCI is a key objective of the service and the SPOT provides consultancy to a range of health professionals and services in the community in order to achieve this objective.

**Fig. 2:**
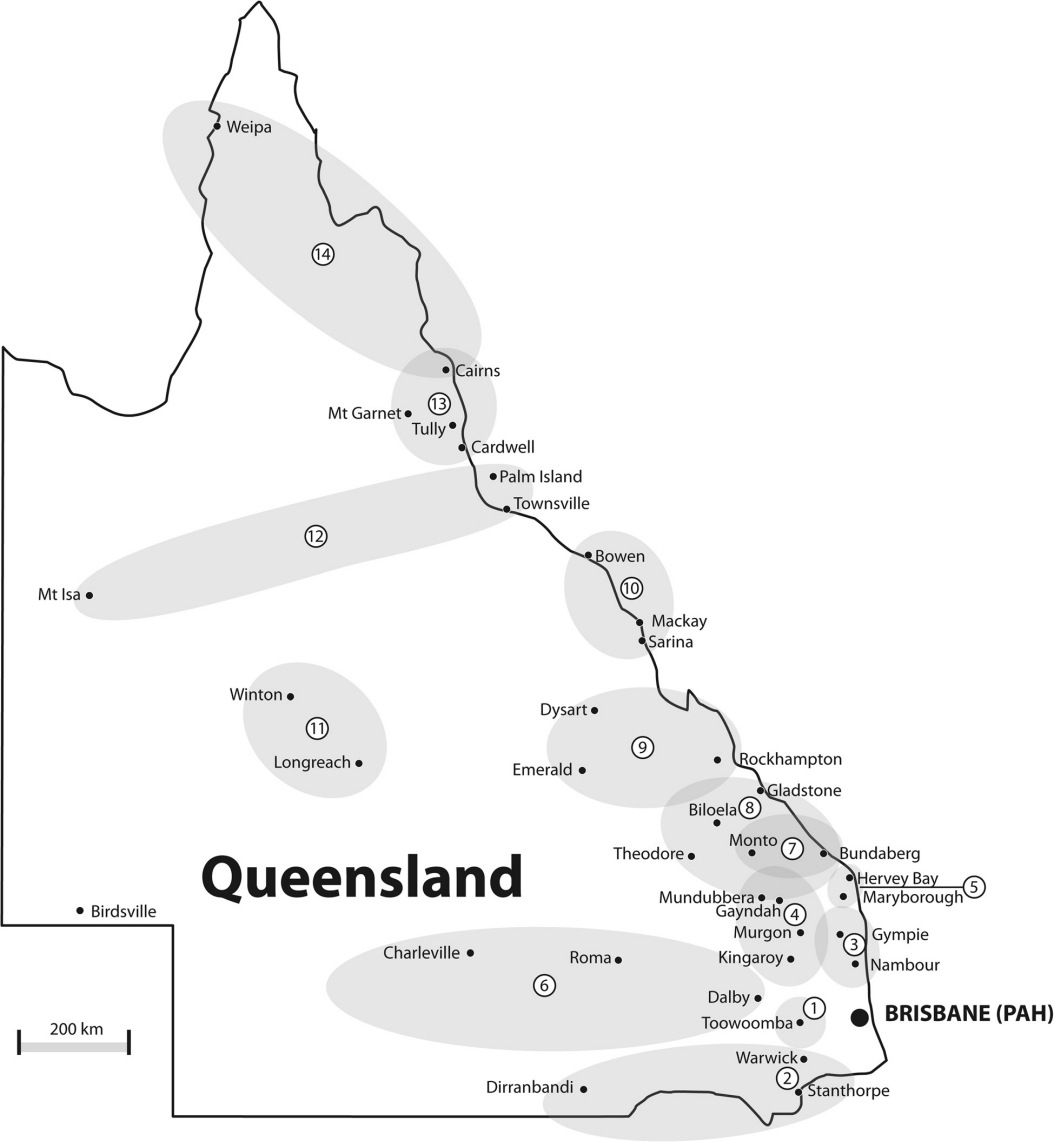
Queensland map showing location of SPOT regional visits

Outreach visits are scheduled by SPOT to 14 locations throughout Queensland, generally once per year, see Table 1 and map shown in Fig. 2.

**Table 1:** SPOT outreach visit locations in Queensland, distance and mode of transport

Outreach regional location	Map reference (see Fig. 2)	Approximate distance from Brisbane (km)	Transport mode
Toowoomba & Pittsworth	1	110–150	Road
Stanthorpe, Warwick and west	2	150–230	Road
Nambour and North to Gympie	3	170–300	Road
Kingaroy, Murgon, Gayndah and Mundubbera	4	220–370	Road
Hervey Bay and Maryborough	5	300–400	Road
Roma, Dalby and west	6	210–480	Road
Bundaberg and west to Monto	7	350–480	Air
Gladstone, Biloela, Theodore and Bundaberg	8	350–540	Air
Rockhampton, and west, Emerald and Dysart	9	640–930	Air
Mackay, south to Sarina and north to Bowen	10	1000–1200	Air
Longreach, Winton and surrounding regions	11	1180–1400	Air
Townsville, Mount Isa and s Surrounding regions (including Palm Island)	12	1350–1840	Air
Cairns and hinterland, south to Cardwell, Tully and Mount Garnett	13	1540–1700	Air
Cairns, and hinterland, north to Cooktown and Cape York	14	1540–2370	Air

Both the SPOT and SIU Outpatient Clinics offer a whole of life service and individuals with SCI are able to refer and re-refer as issues arise along their lifespan.

### Telehealth services

Telehealth is defined as the delivery of health services at a distance through telecommunication technologies. Common examples include communication between patients and therapists via email, telephone or videoconference. Telehealth delivers a range of advantages, particularly for patients living in rural and remote areas. Potential benefits include reduced travel costs, more equitable access to health services and closer engagement with local health services and primary care providers. From a patient’s perspective, the reduced stress, costs and inconvenience of travel away from home for a specialist appointment makes telehealth a favourable alternative [7]. Long distance travel also imposes additional risks associated with transport related accidents. There appears to have been a relatively small amount of experience regarding telehealth applications for people with SCI and only a few reports in the literature mainly from the USA [8]. The systematic literature review of Dorstyn et al. (2014) states that further research is needed to demonstrate the effectiveness and benefits of telehealth for patients with SCI [9].

The aim of this study was to undertake a retrospective audit of current specialist ambulatory services provided by the QSCIS (SIU Outpatient Clinics and SPOT) to better understand the location of patients requiring access to specialist spinal services. These findings may help with the planning of telehealth to support people with SCI living in non-metropolitan areas.

## Methods

### Patient demographics

The general profiles of patients managed by the QSCIS were obtained from the database records maintained by the SPOT. Permission to analyse the data was provided by the clinical service directors. All records were anonymised for patient confidentiality. Referral information included age, gender, cause of injury, type of injury, and place of residence (postcode).

### Service utilisation

A retrospective audit of ambulatory care services provided by the QSCIS during a 6 year period (January 2008 – December 2013) was carried out. SPOT and health service database records were used with the permission of the health service to describe ambulatory services delivered through the Spinal Injuries Unit (SIU) Outpatient Clinics and the Spinal Outreach Team (SPOT). Activity, attendance and referral data was obtained from the central hospital outpatient activity data collection provided by the Metro South Hospital and Health Service and a database managed by SPOT. Activity included specialist consultations provided through the SIU Clinics and SPOT services (telephone support, home visits and outreach visits). Videoconference activity during this period was also reported.

A SPOT occasion of service was defined as any clinical activity that is attributable to a specific patient and is recorded as an occasion of service on the SPOT database and documented in clinical notes. A SPOT occasion of service involved an intervention with a patient, their significant others and service providers, either alone or in combination.

### Place of residence

For all patients who attended an appointment at the SIU clinic or through SPOT, we categorised according to distance from Brisbane. The number of kilometres between place of residence and Brisbane was calculated using postcode and Google Maps. Comparisons were made between patients seen at the SIU clinic and those who received a SPOT service.

Original database reports were prepared for analysis using Microsoft Excel. Records were checked for duplicates and typographical errors. This study was approved by the Metro South Hospital and Health Service, through a formal service level agreement which includes service planning, development and evaluation.

## Results

### Patient demographics

**Fig. 3:**
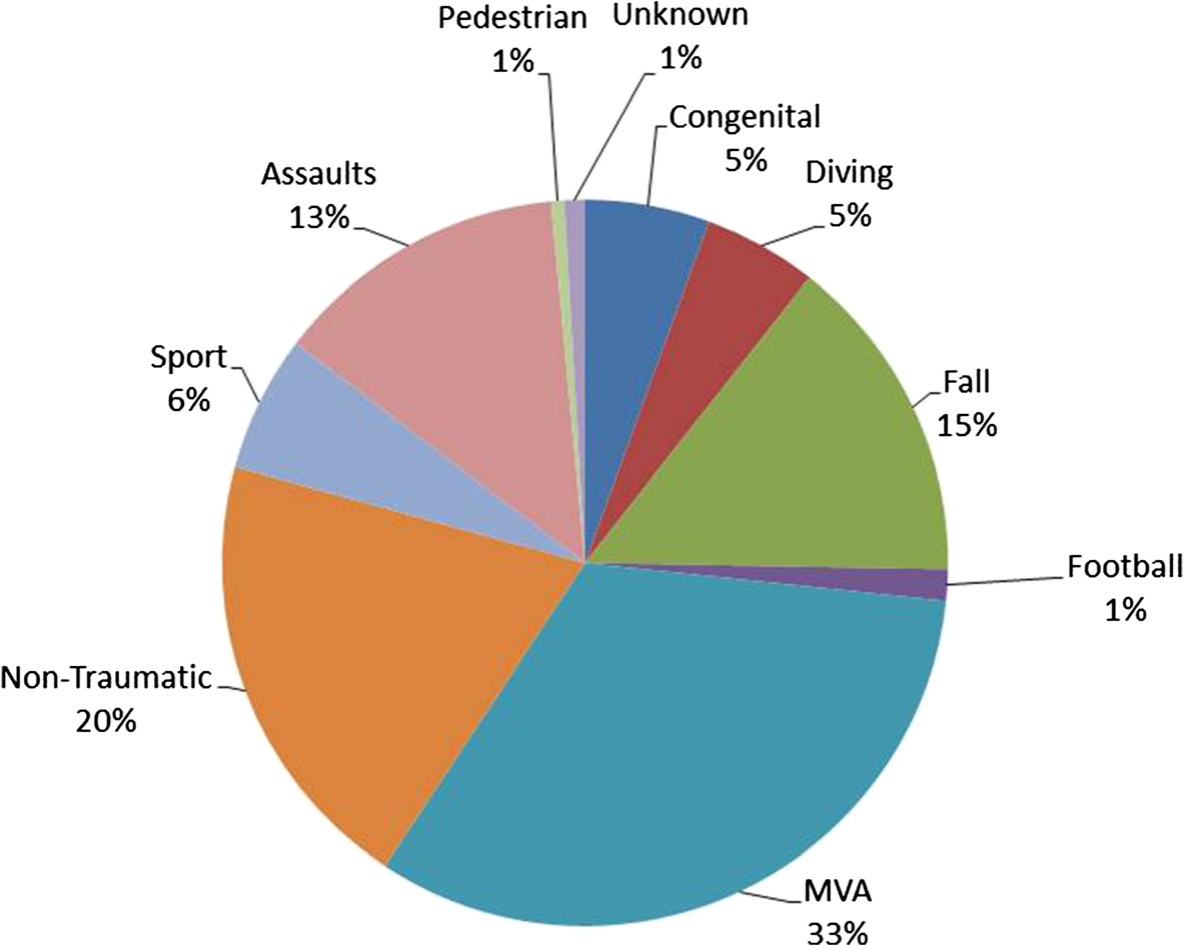
Causes of spinal cord injuries according to SCI referrals to the QSCIS from 1995 to 2013 (*n* = 2073)

A total of 2073 people with SCI, who were still living at the time of data analysis, were referred to the service between 1995 and 2013. 74 % of these were male. The median age was 51 years (IQR 39y-61y). According to level of injury, 53 % had paraplegia level injuries and 41 % had tetraplegia. People with spina bifida accounted for 6 % of those people currently on the database. The leading causes of spinal cord injury were motor vehicle accidents, non-trauma and falls, see Fig. 3.

**Fig. 4:**
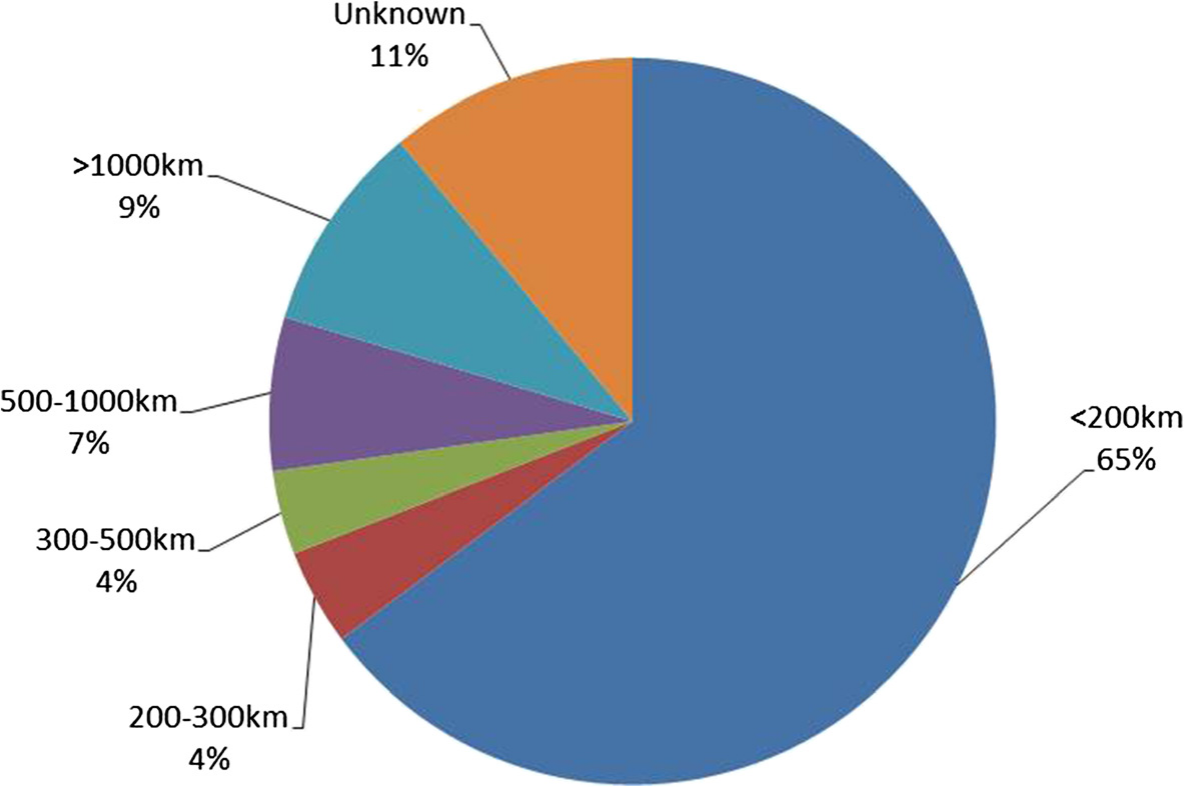
Distance between patients residence and QSCIS - Brisbane (*n* = 2073)

Of all the patients referred to the QSCIS, around one quarter (24 %) lived 200 km or further from Brisbane. Almost two thirds (65 %) lived within 200 km to the hospital in Brisbane, see Fig. 4. The location of 11 % of the patients was unknown due to incomplete data. The average distance for patients treated at the QSCIS was 254 km (range 1 km – 3418 km) from their place of residence.

### Service utilisation

#### SIU Outpatient Clinics

A total of 7513 appointments were held in SIU Outpatient clinics for 1578 people with SCI over the 6 year study period from January 2008 to December 2013. The majority of appointments were for the General SCI clinic (79 %). 15 % of appointments were for the Neuropath Urology Clinic; 3 % attended the Sex and Fertility Clinic; and 3 % attended the Spasticity Clinic. The Did Not Attend (DNA) rate for all SIU appointments over the study period was 17 %.

### Spinal Outreach Team

In the 6 year period, SPOT received 3070 referrals for 1509 individual patients. A total of 43,827 occasions of service were reported during this period. A variety of service delivery methods were utilised including home visiting, hospital and other community location visits, outreach visits, telephone, email and videoconference support. 13 videoconference appointments were provided during the period, with the majority (6) taking place in 2013. Table 2 shows the site occasions of service took place, reported between January 2008 and December 2013.

**Table 2:** Site of SPOT services and volume of activity (January 2008 and December 2013)

Site of service	Occasions of service	Proportion (%)	Duration of services (hours)	Proportion (%)
Agency in community	388	0.89	846	1.9
Patients home	3651	8.33	11,536	25.87
Hospital	574	1.31	1567	3.51
SIU	783	1.79	998	2.24
SPOT offices	38,386	87.59	29,599	66.37
Other/unknown	45	0.10	54	0.12
Total	43,827	100	44,600	100

Occasions of service that take place in either a patient’s home or an agency in the community, including hospitals and the SIU involve direct client contact and represents about one third of all reported activity. Occasions of service that are located in the SPOT office, may also involve direct phone calls and email with clients but also includes consultation with service providers.

**Fig. 5:**
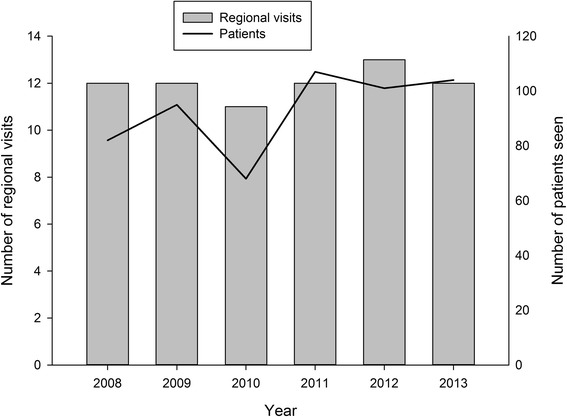
Regional visits and consultations conducted from January 2008 to December 2013

During the 6 year period, 72 outreach visits were provided for a total of 557 patients. Annually, around 100 patients are seen during an outreach visit. The average number of regional outreach clinics held per year was 12, see Fig. 5.

### Place of residence

#### SIU Outpatient Clinics

Of the total number of people who attended appointments at the SIU Outpatient Clinics between January 2008 and December 2013, around 90 % lived within 200 km of Brisbane, see Table 3. Only a small proportion (less than 10 %) of patients who lived further than 200 km from Brisbane attended the outpatient clinic.

**Table 3:** Patients attending SIU appointment – categorised by location

Location	Occasions of service (number)	Proportion (%)
Brisbane region	6773	89.8
(<200 km from QSCIS)		
Outside Brisbane	594	7.9
(>200 km from QSCIS)		
Other	28	0.4
NSW	151	2
Total	7546	100

### Spinal Outreach Team

The SPOT service model includes the option to visit clients in the community either on a daily basis if they are located within a 200 km radius of SPOT’s office in inner Brisbane or as part of an outreach visit if clients are located outside of this 200 km radius.

Two-thirds of all services provided by SPOT involved patients living within 200 km of Brisbane (Table 4).

**Table 4:** Patients receiving SPOT service – categorised by location

Location	Occasions of service (number)	Proportion (%)
Brisbane region	28,806	66
(<200 km from QSCIS office)		
Brisbane region	14,332	33
(>200 km from QSCIS office)		
Other	57	0.13
NSW	393	0.90
Other (international, e.g. PNG)	239	0.55
Total	43,827	100

## Discussion

The QSCIS strives to provide a high quality, equitable service to people with SCI who live in disparate regions of a diverse and geographically vast state. However, anecdotally it is understood that the further away people live, the more logistically difficult it is to access specialist services for follow-up, therefore increasing the potential for inequity of access. The service broadens its reach to patients by offering a range of services in the form of outpatient clinics in Brisbane, outreach visits in regional towns, home visits and telephone support.

This study demonstrated that ambulatory services are provided to patients all throughout Queensland, and that about one third of all patients known to the service live further than 200 km away from Brisbane. Only about 10 % of all attendances at the SIU Outpatient Clinic involved people living further than 200kms from Brisbane. Outreach services provided by SPOT did support a larger proportion (33 %) of patients living further away from Brisbane, mainly by telephone contact, liaison with other health professionals or during physical outreach visits to regional centres.

The number of patients who did not attend (DNA) their appointment at the SIU Outpatient clinic was reported to be 17 %. Whilst one of the most common reported reasons for non-attendance is that patients forget about their appointment, we also suspect the logistical challenges of travelling to Brisbane, costs and availability of (carer) support may also be contributing factors [7]. Non-attendance causes inefficiencies in the health system, and extends the amount of time patients have to wait for an appointment [10].

The QSCIS currently uses a number of methods (with services provided by the SIU and SPOT) to attempt to address inequities caused for people who live at greater distances from the specialist SCI services in Brisbane. Current methods employed to improve the provision of follow-up services and assistance to people in regional, rural and remote centres consist primarily of written and telephone liaison with the individuals themselves, their general practitioners and other local community health and rehabilitation teams. A significant amount of time is also spent engaging with health care providers including rehabilitation medicine and other specialists at other local hospitals where people with SCI are being managed. There is an extensive Urology Database used to assist in managing the annual renal surveillance program in which people with SCI all over the state are sent reminder letters regarding the need to have renal imaging performed. People who live in regional, rural and remote areas have this performed by a local radiology service and the images are reviewed by the SIU Rehabilitation Physicians and Urologist at a weekly Urology Meeting.

More recently (March 2014), formal pre-booked telephone consultations have been introduced. Videoconferencing and face-to-face consultation by SIU medical specialists to distant sites have also occasionally been used but historically this has not been commonplace.

Apart from the SIU OPD, much of the responsibility for provision of ongoing and life-long management and rehabilitation for people with SCI falls to the Spinal Outreach Team. Specialist SCI medical support has traditionally been provided by medical staff from the SIU although more recently (February 2014), dedicated medical staffing for SPOT has also been trialled, in the form of a rehabilitation medicine trainee based in the community SCI teams.

To improve access to specialty SCI services, SPOT utilises a variety of service delivery methods including outreach visits to regional centres and extensive use of telephone and email communication with people with SCI and their local service providers. Videoconferencing is used extensively for training and education of other health professionals and providers.

Despite wide ranging and intensive activity by SPOT, including outreach, telephone, email and use of videoconferencing for education and training activities, this study indicates that the uptake of videoconferencing for clinical assessment and management purposes has not become a commonplace aspect of SPOT activity. The precise explanation for this is unknown but may relate to the extensive and clinically effective use of other telehealth methodologies (email and telephone) in combination with outreach visits, a philosophical preference to provide face-to-face consultation when possible and the historical lack of easy access to videoconferencing facilities for patients at the distant sites. Irrespective of the explanation, the study confirms both a relatively limited use of videoconferencing for clinical purposes in the SIU Outpatient Clinic and SPOT and an important opportunity for future service development in this area.

Research in this area of telehealth and SCI has revealed mixed results. Della Mea (2012) described the use of a web-based specialist support network established for follow up but found that uptake was very low amongst patients and feedback indicated that they felt more comfortable communicating with their GP by telephone [11]. Conversely, a randomised controlled trial by Dallolio et al. compared one group of patients who received standard care whilst the second group received standard care and telemedicine [8]. The study reported much higher satisfaction from the group who had access to telemedicine support. In another study, patients with spinal cord injury (new admissions) were exposed to an intervention upon discharge which involved videoconferencing support, telephone-based support or standard care and found that those supported by videoconference required the least number of hospital admissions [12].

Considering people with spinal cord injury are likely to experience secondary complications following discharge from an acute setting, regular follow up with specialist rehabilitation services and primary care providers such as general practitioners is considered very important. Several studies reported the use of telehealth to prevent, diagnose or treat patients with spinal cord injury with pressure ulcers [13–15]. In one study, videoconferencing was used on a weekly basis to connect patients at home, to monitor and to assess any potential complications. The study by Galea et al. described high satisfaction amongst both patients and caregivers; and a reduced rate of hospitalisations and overall length of stay [16].

A clinical efficacy study of store and forward telehealth, where digital images were used to assess pressure ulcers at a distance was reported by Sarhan et al. [17]. In this study, images were sent to nurses to assess the stage and location of pressure ulcers. Nurses reported high satisfaction associated with the quality of the images collected through this service. Overall agreement regarding the stage and location of pressure ulcers was around 85 %, which showed that a telehealth was a viable means of assessing patients at risk of pressure ulcers [17]. When comparing conventional (face-to-face) assessments, Hill et al. also reported very high agreement for clinical decisions made with the use of digital images for patients with pressure ulcers [18].

Although there is relatively little in the current literature regarding the use of telehealth for people with SCI, it does seem that there are relevant applications which would support and enhance the already extensive services provided by the QSCIS and would be highly beneficial, particularly for people living outside Brisbane and for those requiring regular review.

### Limitations

A limitation of the study was our reliance on multiple databases with differing levels of detail pertaining to their respective services. The choice of outcome measures was dependent on the data available. In regards to the substantial number of occasions of service reported by SPOT in the form of telephone consultations, we were unable to differentiate between occasions of service involving direct client care versus liaison with other service providers.

### Opportunities

There are three potential key areas where telehealth expansion within the QSCIS may be of benefit. The first is; the substitution of current face-to-face medical outpatient appointments with video consultations. This would not only save the patient the need to travel to Brisbane but could be done in partnership with general practitioners and other clinicians in the local community which would encourage better engagement with primary care providers and enhance continuity of care. The second area for telehealth expansion is with increased use of multi-disciplinary SPOT clinical videoconferences which involve SIU medical specialist input as well as SPOT allied health and nursing staff and the third is with SIU medical specialist involvement, by videoconference as part of SPOT outreach visits. In this situation, it is likely that the use of videoconferencing could enhance outreach capabilities, might save some clinicians the need to travel during an outreach visit but also allow a broader range of medical, nursing and allied health practitioners to participate in each outreach visit.

Another possibility is that videoconferencing could provide access to specialist services provided by SIU and SPOT in a more “on demand” manner, outside of the fixed outreach visit schedule and outpatient appointments but this could have significant implications on workforce and resources for both services.

## Conclusion

Telehealth models of care, which promote better engagement with local health service providers (such as general practitioners, nurse practitioners and allied health professionals) should be developed, permitting greater equity of access for people with SCI to the specialist rehabilitation services of QSCIS, whilst reducing the need for long distance travel to and from Brisbane. There is the potential for telehealth, such as videoconferencing, to be used more widely to provide greater flexibility and greater efficiency. Further research is required to determine the feasibility, clinical benefit and economics of telehealth for people with spinal cord injuries.

## Abbreviations

DNA: Did Not Attend
PAH: Princess Alexandra Hospital
QSCIS: Queensland Spinal Cord Injuries Service
SCI: Spinal Cord Injury
SIU: Spinal Injuries Unit
SPOT: Spinal Outreach Team
USA: United States of America
